# Patterns of ethanol intake in male rats with partial dopamine transporter deficiency

**DOI:** 10.1111/gbb.12847

**Published:** 2023-07-17

**Authors:** L. B. Kuiper, J. B. Roberts, P. M. Estave, D. Leo, R. R. Gainetdinov, S. R. Jones

**Affiliations:** ^1^ Department of Physiology and Pharmacology Wake Forest School of Medicine Winston‐Salem North Carolina USA; ^2^ Department of Neurosciences University of Mons Mons Belgium; ^3^ Institute of Translational Biomedicine St. Petersburg State University St. Petersburg Russia; ^4^ St. Petersburg University Hospital St. Petersburg State University St. Petersburg Russia

**Keywords:** alcohol use disorder, binge, dopamine transporter, dopamine, drinking, ethanol, fast‐scan cyclic voltammetry, hypodopaminergia, intermittent access, locomotor activity, novelty, nucleus accumbens, quinine, reuptake, Wistar

## Abstract

Mesolimbic dopamine signaling plays a major role in alcohol and substance use disorders as well as comorbidities such as anxiety and depression. Growing evidence suggests that alcohol drinking is modulated by the function of the dopamine transporter (DAT), which tightly regulates extracellular dopamine concentrations. Adult male rats on a Wistar Han background (DAT+/+) and rats with a partial DAT deletion (DAT+/−) were used in this study. First, using fast‐scan cyclic voltammetry in brain slices containing the nucleus accumbens core from ethanol‐naïve subjects, we measured greater evoked dopamine concentrations and slower dopamine reuptake in DAT+/− rats, consistent with increased dopamine signaling. Next, we measured ethanol drinking using the intermittent access two‐bottle choice paradigm (20% v/v ethanol vs. water) across 5 weeks. DAT+/− rats voluntarily consumed less ethanol during its initial availability (the first 30 min), especially after longer periods of deprivation. In addition, DAT+/− males consumed less ethanol that was adulterated with the bitter tastant quinine. These findings suggest that partial DAT blockade and concomitant increase in brain dopamine levels has potential to reduce drinking and ameliorate alcohol use disorder (AUD).

## Graphical abstract

We investigated patterns of ethanol drinking in male rats with partial deletion of dopamine transporters. Compared to wild‐types, DAT+/− animals do not as readily develop alcohol use disorder (AUD)‐like phenotypes.

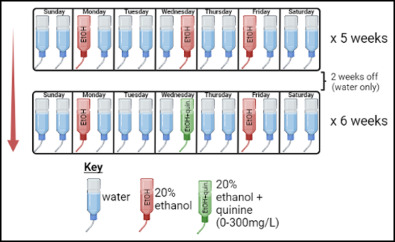

## 1 INTRODUCTION

Alcohol use disorder (AUD) is a leading cause of death (1 in 10 deaths of working‐age adults) and economic burden ($249 billion annually in the United States).^1^ The etiology of AUD is influenced by multiple genes, environmental factors and exposure to alcohol itself.^2^, ^3^ In recent years, it has become clear that initial sensitivity to alcohol's reinforcing and sedating effects modulate early drinking behaviors, but withdrawal‐related anxiety and other negative affective states are a major driver of continued use and relapse to alcohol use in AUD. Further understanding of the neurobiological bases of alcohol‐related behaviors will help guide efforts to mitigate the personal and societal impact of AUD.

Multiple neurotransmitter systems are implicated in substance use disorders, including AUD. Dopamine (DA) signaling in the mesocorticolimbic system guides motivated behaviors necessary for homeostasis, ensuring that behavior is appropriately directed towards acquiring resources and avoiding threats. As such, DA has been implicated in the motivating and reinforcing effects of all known misused substances, including alcohol. This system is vulnerable, however, to disruption by alcohol exposure, contributing to pathological states characterized by the compulsive pursuit of rewarding stimuli despite known negative consequences. In addition, AUD is highly comorbid with other psychiatric diseases related to DAergic pathology, including attention‐deficit hyperactivity disorder,^4^ major depressive disorder,^5^ bipolar disorder,^6^ and schizophrenia.^7^ Further research is needed to understand the mechanisms by which DAergic pathologies promote the development of AUD.

DAergic signaling in the brain is dynamically regulated by the interaction of DA release and reuptake, which are in turn subject to modulation by numerous receptors and intracellular effectors reviewed in.^8^, ^9^, ^10^, ^11^, ^12^ The primary mechanism by which signaling is terminated is through the rapid removal of DA from the synapse via DA transporters (DATs) located presynaptically on DAergic neurons. The role of DATs in DAergic transmission, as well as the pharmacological mechanisms of acute drug actions, has been studied extensively using mice with genetically altered DAT function, such as DAT knock‐out (DAT−/−) mice.^13^, ^14^, ^15^, ^16^ These mice have also been used to study the neurobiology underlying hyperactivity, attention, compulsive behavior, anxiety and Parkinson's disease.^17^, ^18^, ^19^, ^20^, ^21^, ^22^ Recently, a line of DAT knock‐out rats was generated using zinc‐finger technology to silence the *SLC6A3* gene which encodes the DAT,^23^ expanding opportunities in areas of neurobiological research for which rat models are uniquely useful. Of particular interest for the present study are the heterozygous (DAT+/−) offspring. These animals possess only one copy of the normal allele, resulting in partial DAT deletion. Using fast‐scan cyclic voltammetry, Leo and colleagues demonstrated that DAT+/− rats exhibit slower DA reuptake than DAT+/+ littermates, but faster reuptake than DAT−/− littermates.^23^ This “intermediate phenotype” is also evident when examining DA levels in the striatum using microdialysis.^23^, ^24^ This, combined with similar findings in mice, demonstrates that decreased DAT expression in DAT+/− animals leads to hyperdopaminergia, or increased dopamine tone. Hypodopaminergia (decreased dopamine tone), in contrast, has been posited to underlie negative affect, or hyperkatifeia. Increased drinking ensues as a means of alleviating this aversive state.^25^ We therefore hypothesize that increased levels of DA in DAT+/− rats will be protective against AUD‐like patterns of ethanol intake.

The goal of the present study was to characterize patterns of voluntary ethanol intake in DAT+/− rats, compared with wild‐type (DAT+/+) controls, given intermittent access to 20% ethanol. This paradigm has been shown to gradually produce escalated ethanol intake and preference over a period of weeks in wild type rats.^26^ The effect of partial DAT deletion on aversion‐resistant ethanol drinking was also examined.

## 2 MATERIALS AND METHODS

All animal procedures were approved by the Institutional Animal Care and Use Committee at Wake Forest School of Medicine and adhere to the National Institutes of Health guidelines. Experiments were performed in Association for Assessment and Accreditation of Laboratory Animal Care accredited facilities.

### 2.1 Animals

All experimental rats were from a breeding colony of Wistar‐Han DAT knockout rats within our facility, originally described in.^23^ Adult male wild‐type and heterozygous rats (age matched, ca. 17 weeks of age) were from various types of parental pairings (Table S1). Animals with the same parental genotype were either littermates or originated from separate parents with the same genotype. The type of parental pairing or the genotype of the rearing mother had no apparent effect on any of the outcomes measured. Full DAT knockout (DAT−/−) offspring were not used in this study. Animals were given access to food and two water bottles ad libitum and were maintained on a reversed light cycle (12 h on, 12 h off), with lights off at 0800 h. Two weeks before the onset of the alcohol drinking procedures, animals were housed individually.

### 2.2 Fast‐scan cyclic voltammetry

Ethanol‐naïve subjects (3 DAT+/+ and 5 DAT+/−) were anesthetized using isoflurane vapor and rapidly decapitated. Brains were quickly removed and submerged in oxygenated (95% O_2_/5% CO_2_) ice‐cold artificial cerebral spinal fluid (aCSF, 126 mM NaCl, 1.2 mM NaH_2_PO_4_, 2.4 mM CaCl_2_, 1.2 mM MgCl_2_, 25 mM NaHCO_3_, 11 mM glucose, 0.4 mM L ascorbic acid). Coronal brain slices (400 μm thick, 4–6 slices per animal) containing the nucleus accumbens core were obtained using a vibratome (Leica VT1000S) and transferred to a recording chamber perfused with oxygenated aCSF (32°C) at 1 mL/min. After equilibrating for approximately 30 min, a microelectrode containing a single carbon fiber (pulled through a glass capillary tube and trimmed to 120–150 μm in length, 7 μm diameter) and a bipolar stimulating electrode were placed in the nucleus accumbens core at a distance of approximately 100 μm between electrodes. The carbon fiber electrode penetrated the surface of the slice at approximately 75 μm in depth. A triangular waveform (−0.4 to +1.2 to −0.4 V vs. Ag/AgCl, 400 V/s) was applied to the carbon fiber electrode, and the resulting background was subtracted from the recorded signal. DA was evoked by a single, rectangular, electrical pulse (750 μA, 2 ms, uniphasic) once every 5 min. DA was measured every 100 ms and recorded during 15‐s collection windows. Stable DA collections were used for analysis: stability was reached after no increase or decrease in the maximum amplitude of DA current was detected across five collections, usually after 15–20 collections or approximately 90 min. Each electrode was calibrated with 3 μM DA in aCSF (room temp.) using a flow‐injection system immediately after recording was completed. Stable baseline DA signals were modeled using Michaelis–Menten kinetics and exponential curve‐fitting (peak/decay analysis) using Demon analysis software.^27^

### 2.3 Locomotor activity

The following procedure took place under dim red light (0 lux) during the first half of the dark (active) cycle. Rats (6 DAT+/+ and 8 DAT+/−) were acclimated to the experimental room for 15 min in their home cage. Each animal was then placed in the center of a novel open field (Med‐Associates, Fairfax, Vermont; dimensions 43 × 43 × 30 cm) equipped with a 16 × 16 photobeam array connected to software (Activity Monitor, Med‐Associates) which recorded activity for 30 min. Recording chambers were cleaned using soap and water and allowed to thoroughly dry between animals.

### 2.4 Intermittent access two‐bottle choice

All drinking procedures began in the first half of the animals' active (dark) cycle (approx. 3 h after lights off). Each Monday, Wednesday and Friday, rats (14 DAT+/+ and 16 DAT+/−) were given a choice between 20% ethanol (diluted in tap water) and tap water in 50 mL conical tubes. Bottles were weighed to measure liquid consumption after the first 30 min of access and again after 24 h. Two experimental bottles were placed in an empty cage and were handled similarly to those given to the rats to control for spillage/drip. Between drinking sessions, conical tubes were replaced with two regular housing water bottles containing tap water.

### 2.5 Quinine adulteration

After 2 weeks of forced abstinence from ethanol drinking, a subset of rats (8 DAT+/+ and 8 DAT+/−) from the intermittent access two‐bottle choice experiment continued to the quinine adulteration phase of the study, which took place across 6 additional weeks on an intermittent schedule identical to the intermittent‐access two‐bottle choice paradigm. On Wednesdays during these weeks, the 20% ethanol was adulterated with varying quinine concentrations (0, 10, 30, 100, 300 mg/L; quinine hydrochloride, Sigma‐Aldrich, St Louis, MO) in 20% ethanol. No quinine was added during the first and last week to serve as baseline comparisons. Drip and spillage control were conducted as described above.

### 2.6 Data management, inclusion and exclusion

**FIGURE 1:**
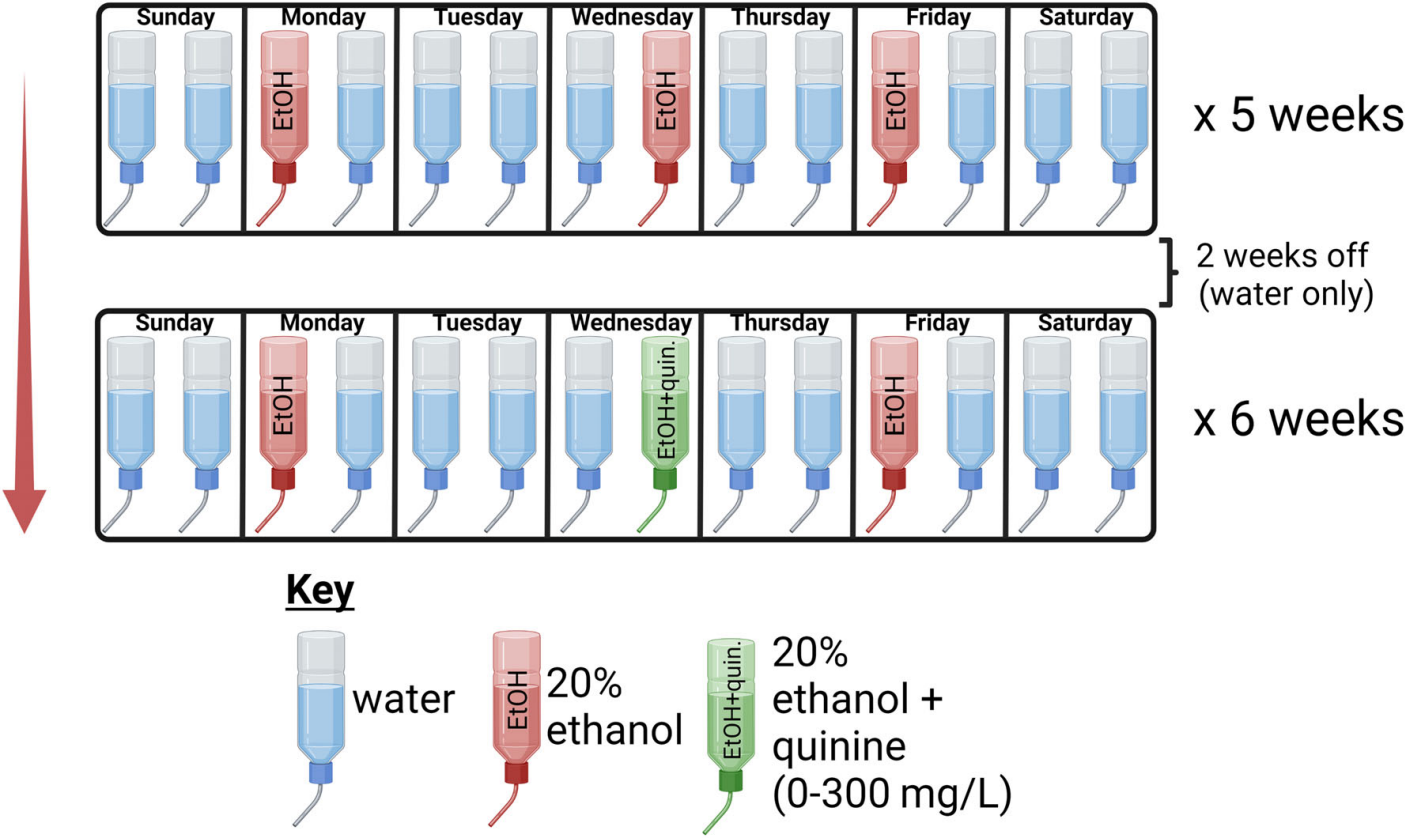
Single pulse evoked [DA]_e_ and reuptake in the nucleus accumbens core of DAT+/+ and DAT+/− males measured by FSCV. (A) Averaged mean (dark shading) ± SEM (light shading) evoked DA (measured every 0.1 s) across time during final stable collection under baseline conditions; representative color plots are shown below, depicting the voltammetric current (shown on scale to right) as color along the *z*‐axis, plotted against the applied potential on the *y*‐axis and time on the *x*‐axis. (B) Mean ± SEM peak evoked DA concentration and (C, D) reuptake, expressed as (C) *V*_max_ (maximum rate of DA reuptake) and (D) half‐life (time required for half of DA clearance); *n* = 3 animals, 13 slices DAT+/+, and 5 animals, 19 slices DAT+/− ****p < 0.0001, unpaired *t*‐test.

Fast‐scan cyclic voltammetry (FSCV) data (Figure 1) were excluded from final analysis for only the following a priori reasons: failure of baseline dopamine signals to reach stability (DAT+/+: 1 slice, DAT+/−: 2 slices), faulty or excessively noisy recording electrodes (two slices from each group), inexplicable environmental noise (DAT+/+: 1 slice). Animals were excluded from analysis of drinking experiments due to near‐complete lack of ethanol drinking (3 DAT+/+ animals), and one drinking animal was removed as a statistically significant outlier (Grubbs test, *p* < 0.05). Ethanol, water and fluid intake from the second day of intermittent‐access two bottle choice was excluded from analysis of a subset of animals (*n* = 9/group) due to unintentional overnight water deprivation prior to the session, which led to unusually high water intake. All data are available on request from the authors.

### 2.7 Statistical analysis

Two way repeated‐measures analysis of variance (RM ANOVA) was used to analyze liquid (ethanol, water) intake and ethanol preference across intermittent access two‐bottle choice sessions. Unpaired two‐tailed *t*‐tests were used to analyze all FSCV measurements, the slope of linear regressions calculated for each animal's ethanol intake across drinking sessions, total ethanol intake, ethanol intake and preference during quinine adulteration. All data were analyzed and graphed in GraphPad Prism 9 software. Data are presented as mean ± SEM, with a significance threshold of *p* < 0.05.

## 3 RESULTS

### 3.1 Greater evoked dopamine concentration and slower reuptake in the nucleus accumbens core of DAT+/− males

DA kinetics were evaluated previously in brain slices containing the dorsal striatum from DAT+/+ and DAT+/− male rats.^23^ These data showed no differences in evoked DA concentration between the two groups, but there was slower reuptake in DAT+/− rats. The ventral striatum, which includes nucleus accumbens core and shell, is of particular interest when evaluating reward‐related learning and motivation.^10^, ^28^ Here, in a group of alcohol‐naïve subjects (3–5 animals and 13–19 slices/group), our findings demonstrate that in the nucleus accumbens core, DA concentration evoked by single electrical pulses is greater in slices from DAT+/− males than those of DAT+/+ males (mean ± SEM DA concentration: 0.933 ± 0.358 μM DAT+/+, 1.267 ± 0.401 μM DAT+/−; *t* (30) = 2.412, *p* = 0.0222, Figure 1B). Additionally, reuptake rate (*V*_max_) was significantly slower in DAT+/− males (mean ± SEM reuptake values: 3.493 ± 1.049 μM/s DAT+/+, 1.477 ± 0.565 μM/s DAT+/−; *t* (30) = 7.047, *p* < 0.0001, Figure 1C). As expected, DA clearance was significantly slower in DAT+/− slices (mean ± SEM half‐life values: 0.267 ± 0.037 s DAT+/+, 0.498 ± 0.089 s DAT+/−; *t* (30) = 8.848, *p* < 0.0001, Figure 1D).

### 3.2 Locomotor response to novel open field is greater in DAT+/− than DAT+/+ males

**FIGURE 2:**
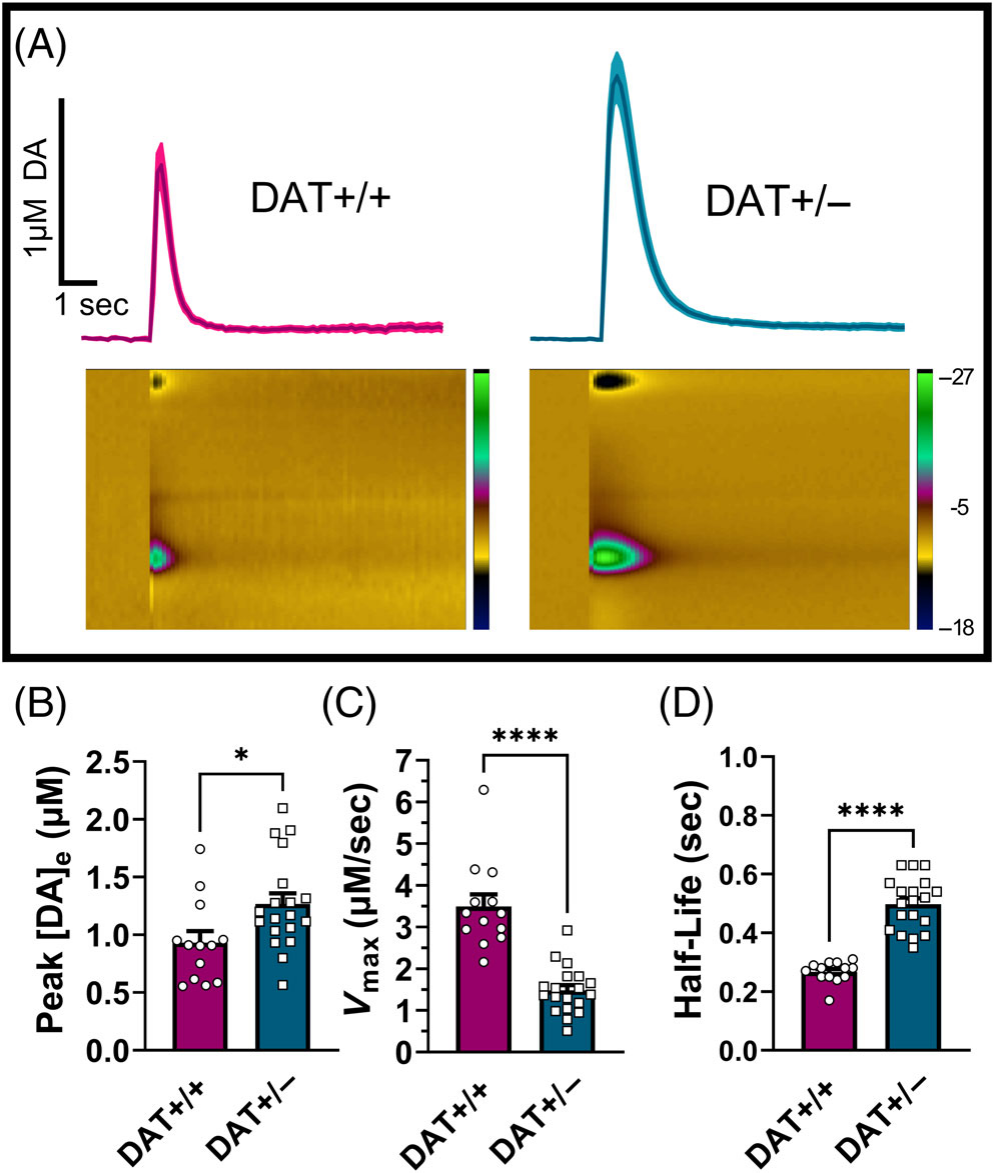
Horizontal activity (distance traveled) and anxiety‐like behavior in a novel open field. (A) Mean ± SEM distance traveled (m) per minute (A) and total (B) during 30‐min session. (C) Mean ± SEM percent of time spent on perimeter of novel open field. *p < 0.05 (unpaired *t*‐test) **p < 0.01 (2‐way RM ANOVA panel A, unpaired *t*‐test panels B and C). (D, E) Traces of beam breaks (horizontal activity) of one representative animal from each group.

Response to novelty (locomotor activity in a novel open field) was assessed in a subset of males (*n* = 6–8/group) prior to the onset of voluntary ethanol drinking. DAT+/− males exhibited a significantly increased response to novelty (i.e., traveled a greater horizontal distance) than DAT +/+ males during the 30‐min locomotor activity session (*F* (1, 16) = 9.665, *p* = 0.0025, Figure 2A; total distance traveled *t* (16) = 3.572, *p* = 0.0025, Figure 2B). Interestingly, DAT+/− males spent a lesser proportion of time on the perimeter of the arena (a putative measure of anxiety‐like behavior) than DAT+/+ males (*t* (16) = 2.165, *p* = 0.046, Figure 2C). This observation is consistent with previous reports showing decreased anxiety‐like behavior of DAT+/− rats in several experimental protocols.^29^, ^30^, ^31^

### 3.3 Patterns of voluntary ethanol consumption differ in DAT+/+ and DAT+/− males

**FIGURE 3:**
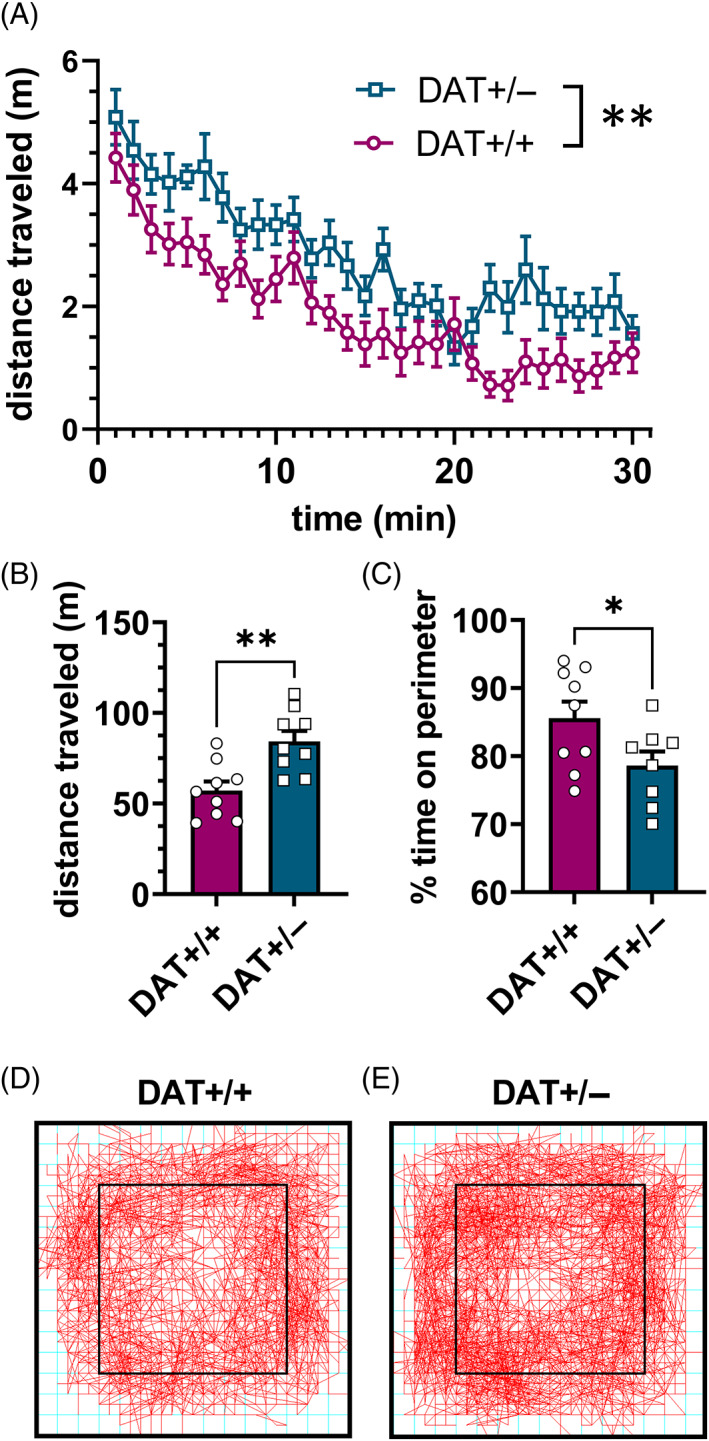
Experimental timeline for voluntary drinking study. Created with BioRender.com.

**FIGURE 4:**
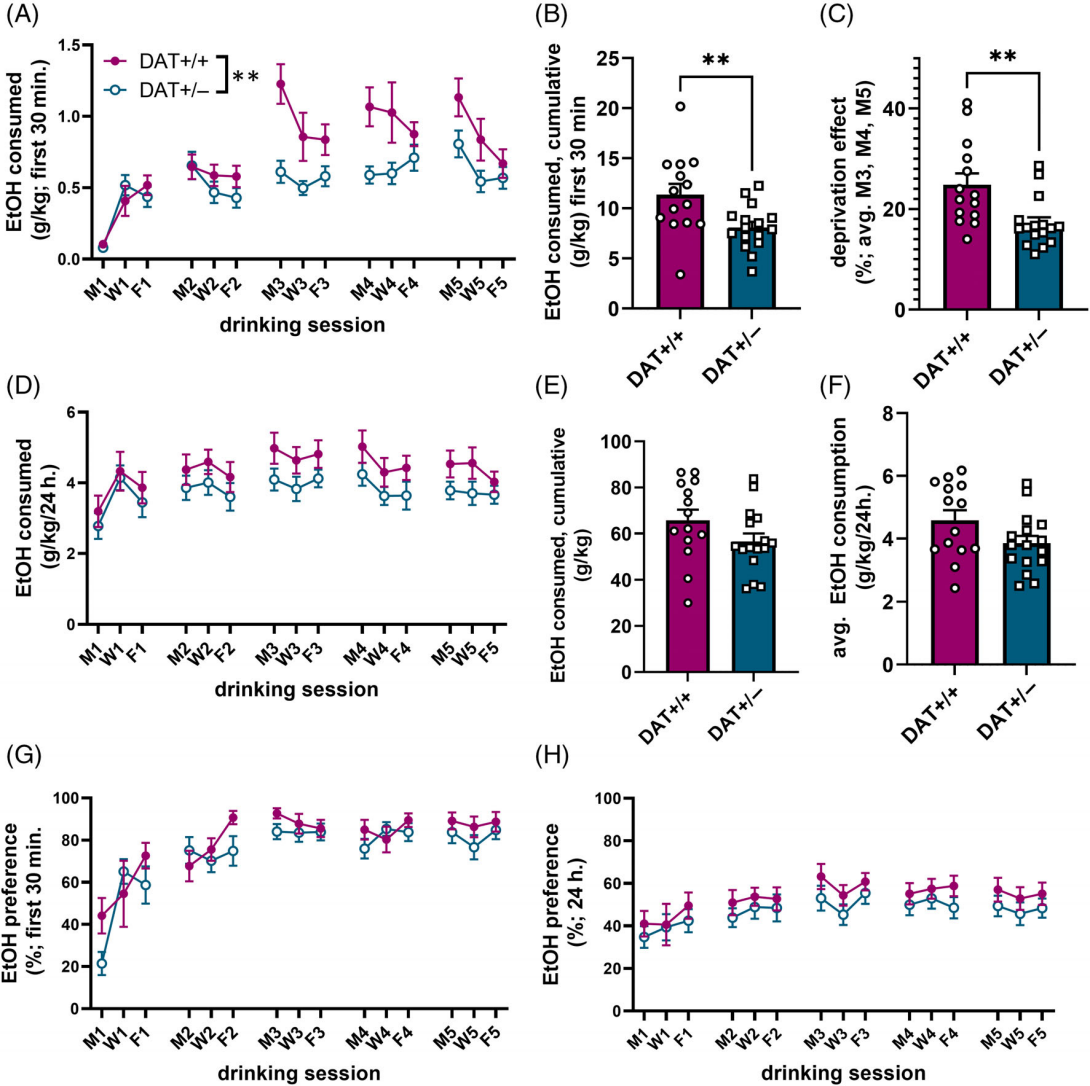
Altered pattern of ethanol consumption in DAT+/− males during first 30 min of intermittent access two‐bottle choice sessions. (A) Mean ± SEM voluntary consumption of 20% ethanol during the first 30 min of each session. (B) Ethanol (EtOH) intake during the first 30 min, cumulative across all sessions. (C) Deprivation effect: On Mondays, after extended period of EtOH deprivation over the weekend, the amount of EtOH consumed in the first 30 min. divided by total; averaged over Mondays 3–5. (D) Mean ± SEM voluntary consumption of 20% ethanol during each session. (E) Ethanol (EtOH) intake (g/kg), cumulative across all sessions. (F) Average EtOH consumption across weeks 3–5. (G) EtOH preference (EtOH consumed divided by total fluid consumed) during first 30 min of each session. (H) EtOH preference (EtOH consumed divided by total fluid consumed) over entire session. **p < 0.01 (RM two‐way ANOVA and unpaired *t*‐test).

A timeline of drinking procedures is provided in Figure 3. Animals (*n* = 14–16/group) were given intermittent access (Monday, Wednesday and Friday) to 20% ethanol and tap water for 5 consecutive weeks, and ethanol consumption was measured after 30 min (termed “binge‐like” consumption^32^) and after the full 24 h of each access period. Two‐way RM ANOVA for the first 30 min of voluntary consumption across sessions revealed main effects of drinking session (*F* (6.534,183.0) = 2.910, *p* < 0.0001), genotype (*F* (1, 28) = 7.971, *p* = 0.0087), and a significant interaction between genotype and session (*F* (14,392) = 2.910, *p* = 0.0003, Figure 4A). In total, DAT+/+ males consumed more ethanol in the first 30 min of all sessions than DAT+/− males (*t* (28) = 2.823, *p* = 0.0087; Figure 4B). By the third week, DAT+/+ rats exhibited an alcohol deprivation effect, that is, greater ethanol consumption during the initial 30 min of ethanol availability on Mondays, following an extended (48 vs. 24 h) alcohol abstinence period between Friday and Monday sessions. A significant difference between groups was found when comparing the proportion of ethanol consumed during the first 30 min of the first (Monday) session of the week, averaged across weeks 3, 4 and 5 (*t* (28) = 3.135, *p* = 0.004, Figure 4C). Importantly, this effect was not present during Wednesday and Friday sessions (Figure S1). Groups did not differ in the total amount of ethanol consumed across all sessions (Figure 4D). Data for water and total fluid intake are shown in Figure S2. In contrast to the effects of DAT reduction on binge‐like ethanol drinking behavior, no differences between groups were found in the pattern of ethanol consumption or the total amount of ethanol consumed across the 5 weeks of intermittent choice access (Figure 4D–F). Groups did not differ in preference for alcohol (Figure 4G,H), likely because ethanol comprised the vast majority of fluid intake in both groups.

### 3.4 Locomotor response in novel open field predicts deprivation effect

**FIGURE 5:**
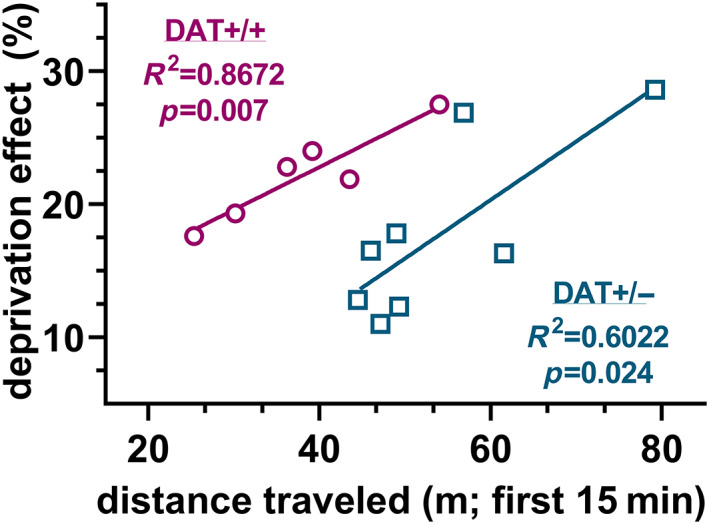
Locomotor response to novelty predicts future binge‐like drinking (see Figure 3A for full analysis of locomotor activity; see Figure 4 for full analysis of EtOH drinking and Figure 4C description of the “deprivation effect”).

The locomotor response to novelty (*x*‐axis; shown in Figure 2) predicted future drinking behavior (*y*‐axis; shown in Figure 4C) in both groups, and assessment of correlation coefficients suggests a weaker relationship between novelty response and binge‐like drinking in DAT+/− males compared with that of DAT+/+ males (*R* ^2^ = 0.60 and 0.87, respectively; Figure 5).

### 3.5 Differences in consumption of alcohol adulterated with quinine between DAT+/+ and DAT+/− males

**FIGURE 6:**
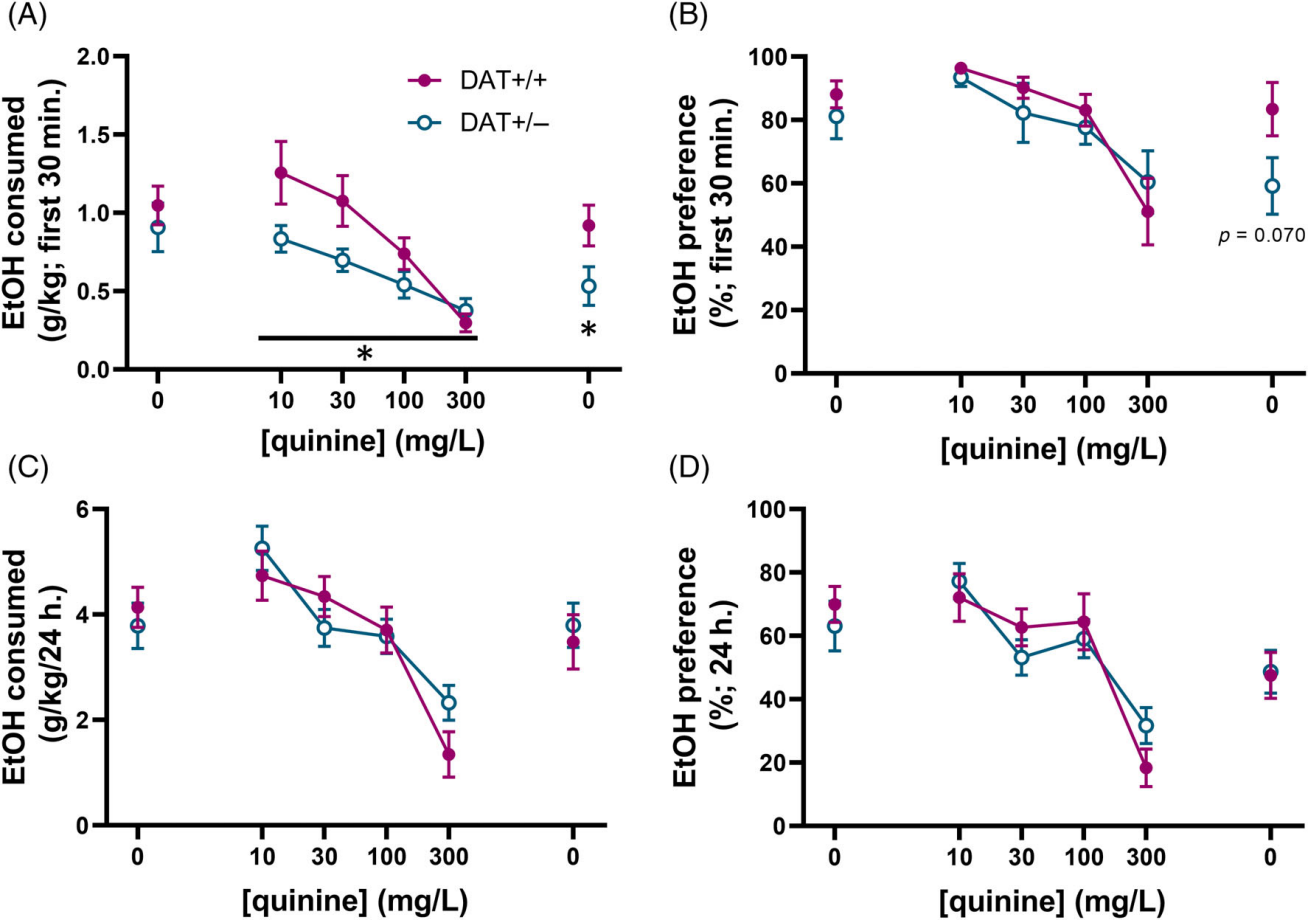
Differences in consumption of alcohol adulterated with quinine between DAT+/+ and DAT+/− males. (A) Mean ± SEM voluntary consumption (during first 30 min) of 20% ethanol adulterated with 0–300 mg/L quinine. (B) Mean ± SEM percent ethanol preference (during first 30 minutes). (C) Mean ± SEM voluntary consumption (during full 24 h). (D) Mean ± SEM percent ethanol preference (during full 24 h). *p < 0.05 (RM two‐way ANOVA and unpaired *t*‐test).

Consumption of ethanol adulterated with the bitter tastant quinine was measured in a subset of males (*n* = 8/group) 2 weeks after the completion of intermittent access two‐bottle choice. Repeated measures RM ANOVA revealed a main effect of quinine concentration (*F* (1.884,26.37) = 23.46, *p* < 0.001) and an interaction effect (quinine concentration × genotype) on binge‐like ethanol consumption (*F* (3, 42) = 3.167, *p* = 0.034; Figure 6A). Interestingly, when given a choice between unadulterated 20% ethanol and water the week following quinine adulteration, DAT+/+ males consumed more ethanol than DAT+/− males (*t* (14) = 2.147, *p* = 0.0498, Figure 6A). Group comparison of preference for unadulterated ethanol the week following quinine adulteration did not reach statistical significance (*t* (14) = 1.965, *p* = 0.070; Figure 6B). There were no differences in ethanol consumption nor preference during the full 24 h of the sessions during which ethanol was adulterated with quinine (Figure 6C,D).

## 4 DISCUSSION

Here, we demonstrate that adult DAT+/− males exhibit increased evoked DA concentration and slower DA reuptake in nucleus accumbens core slice preparations. We also demonstrate that partial DAT deletion reduces AUD‐like phenotypes. When subjects were allowed to voluntarily consume ethanol in an intermittent fashion—a well‐established model that produces robust escalation of ethanol intake^26^—males heterozygous for the expression of the DAT gene exhibited less binge‐like ethanol consumption and “compulsive” ethanol intake.

The finding of slower DA reuptake in the nucleus accumbens core provides additional functional evidence of elevated DA tone in DAT+/− animals, a relationship that has been extensively demonstrated previously in mice^33^, ^34^ and is further evidenced by increased locomotor activity shown in this study. It has been shown previously by our laboratory and others that increased DAT function, and thus lowered DA tone, is associated with increases in ethanol consumption,^35^ which in turn elevates DA levels.^36^ It has also been shown that agents which increase synaptic levels of DA decrease ethanol intake in animal models.^37^ Importantly, acute ethanol withdrawal further drives decreases in DA tone.^36^ The intermittent access model employed in our study includes regular periods of deprivation, and significant increases in binge‐like ethanol consumption are especially evident after longer (48 vs. 24 h) periods of deprivation, which is consistent with the “alcohol deprivation effect” (for further discussion on this topic, see^38^). A key finding of the current study is that the alcohol deprivation effect is absent in DAT+/− males, suggesting that elevated DA tone is protective against binge‐like alcohol consumption and abstinence‐induced augmentation of drinking. This may also indicate that DAT+/− animals are resistant to neuroplasticity associated with escalated drinking. Further, when quinine was added within drinking epochs, drinking patterns consistent with “aversion‐resistant” drinking were evident in the DAT+/− animals. For comparison, when animals exposed long‐term to ethanol show less aversion to quinine adulteration at the low, but not high, concentrations of quinine.^39^ Here, partial DAT deletion led to greater sensitivity to the aversive effects of quinine, further indicating the presence of a protective mechanism (e.g., elevated DA tone).

We show in the present study that locomotor response to novelty predicts drinking after 48 h of deprivation (here termed the “deprivation effect,” see Figure 4C). Response to novelty in rodents is well established as a predictor of psychostimulant self‐administration,^40^, ^41^, ^42^ such that “high responders” acquire self‐administration of drugs more rapidly than “low responders.” Likewise, in humans, measures of sensation seeking are linked with drug and alcohol use.^43^, ^44^ Prior work in DAT+/− rats demonstrate in home cage, or in long or repeated sessions in the open field, that general locomotor activity does not differ from DAT+/+ animals.^23^, ^45^ Here, we focus on initial locomotor activity in a novel arena, and we provide a direct link between response to novelty and ethanol consumption in rodents. Interestingly, the relationship between the locomotor response to a novel environment and binge‐like drinking, though significant, appears to be weaker in DAT+/− males than in wild‐type counterparts. We therefore postulate that when limbic DA levels are greater, other factors in addition to those tied to novelty‐seeking become more likely to explain the propensity to binge drink. Future experiments will aim to investigate the neurobiological correlates of drinking when DA levels are altered.

In the present study, there was no apparent effect of partial DAT deletion on preference for ethanol over water, and both groups readily formed and maintained a similar preference for 20% ethanol. The two‐bottle choice method employed in this study, despite having high face validity, does not allow the experimenter to measure liquid consumption over time at a high resolution. Therefore, latency to drink and lengths of drinking bouts, for example, are unknown. Knowing whether DAT+/− males reach satiety in a shorter period of time, with longer epochs between drinking bouts, or whether drinking is evenly distributed across sessions would provide further insight into whether these animals exhibit altered motivation to consume ethanol. A future study utilizing home–cage bottles outfitted with a lickometer^46^, ^47^ in addition to operant ethanol self‐administration procedures^48^ would achieve this goal and allow for more precise measurement of patterns of appetitive (i.e., craving) and consummatory behaviors. Further experimentation is also needed to determine whether DAT+/− males are differentially sensitive to the acute effects of ethanol (metabolic, locomotor/righting reflex, rewarding) and to determine whether DAT+/− males are more resistant to the tolerance‐producing effects of ethanol. While the paradigm used in this study is useful to model human‐like ethanol intake patterns through repeated cycles of drinking with short periods of deprivation, testing whether partial DAT deletion affects ethanol intake under continuous access conditions, in dependence states such as those produced by chronic intermittent ethanol vapor exposure, or in binge‐like ethanol consumption paradigms, that is, drinking in the dark^49^ would provide a more complete understanding of drinking patterns exhibited in rats with genetic DAT deficiency.

An obvious limitation of this study is the lack of female subjects. Studies requiring in‐house breeding of experimental subjects requires an exceptional amount of planning, time and housing space within the vivarium. Because of this, we decided that it would be the most efficient use of resources to conduct these studies using one sex at a time in order to include adequate sample sizes (in replicate cohorts) in a reasonable amount of time.

## 5 CONCLUSION

To conclude, our results provide further evidence that DA signaling plays a key role in alcohol‐related behaviors and, specifically, that increased DA signaling may reduce the motivation to consume alcohol. Further studies can take advantage of this model of DAT deficiency to develop targeted therapies for AUD and other related psychiatric disorders.

## Supporting information


**Data S1** Supporting Information

## Data Availability

All data are available on request from the authors.
