# Supplementary material for: Patterns of ethanol intake in male rats with partial dopamine transporter deficiency
Source: Genes Brain Behav. 2023 Jul 17;22(6):e12847. doi: 10.1111/gbb.12847 (PMC10733570; doi:10.1111/gbb.12847)
Supplement: Supplementary file 1 — Data S1 Supporting Information [file GBB-22-e12847-s001.docx]

**Supporting Information**

**­Patterns of ethanol intake in male rats with dopamine transporter deficiency**

L.B. Kuiper, J.B. Roberts, P.M. Estave, D. Leo, R.R. Gainetdinov, S.R. Jones

Contents:

Supplementary Table 1

Supplementary Figures 1-2

|  | **Dam:** | **DAT+/-** | **DAT+/-** | **DAT+/+** | **DAT+/-** |
| --- | --- | --- | --- | --- | --- |
|  | **Sire:** | **DAT+/-** | **DAT+/+** | **DAT+/-** | **DAT-/-** |
| Voltammetry study | **DAT+/+** | 3/3 | -- | -- | -- |
|  | **DAT+/-** | 5/5 | -- | -- | -- |
| Drinking study | **DAT+/+** | 4/14 | 8/14 | 2/14 | -- |
|  | **DAT+/-** | 1/16 | 9/16 | 1/16 | 5/16 |

**Supplementary Table 1** | Parental pairings of offspring used in each experiment.

**Supplementary Figure 1** | Lack of alcohol deprivation effect on Wednesdays and Fridays. M3-5: Mondays of weeks 3, 4 and 5; W3-5: Wednesdays of weeks 3, 4 and 5; F3-5: Fridays of weeks 3, 4 and 5. Values from each animal were averaged across the three weeks, data are displayed here as group mean ± SEM. ** p < 0.01 (same comparison as shown in Fig. 4C)

**Supplementary Figure 2** | (A,B) Mean±SEM water (A) and fluid (B; EtOH + water) consumption during the first 30 min. of each intermittent access two-bottle choice session. (C,D) Mean±SEM water (C) and fluid (D; EtOH + water) consumption during each session. ** p < 0.01, *** p < 0.001
